# P-713. Epidemiology and Risk Factors for Community-Acquired Pneumonia Among Late-Stage Older Adults: A Retrospective Study Using the Japanese Health Insurance Database in Fukui Prefecture, Japan

**DOI:** 10.1093/ofid/ofaf695.925

**Published:** 2026-01-11

**Authors:** Hisato Yoshida, Akiko Matsunaga, Naohiro Konoshita, Hitoshi Yoshimura, Ippei Sakamaki, Masamichi Ikawa, Masayuki Nigo

**Affiliations:** Houston Methodist Research Institute, Yoshida-gun, Fukui, Japan; University of Fukui, Fukui, Fukui, Japan; University of Fukui, Fukui, Fukui, Japan; University of Fukui, Fukui, Fukui, Japan; University of Fukui, Fukui, Fukui, Japan; University of Fukui, Fukui, Fukui, Japan; Houston Methodist Hospital, Houston, Texas

## Abstract

**Background:**

Pulmonary infections remain a major cause of morbidity and mortality, particularly among older adults in Japan. As the aging population continues to grow, understanding the epidemiology and risk factors associated with pulmonary infections is crucial for developing effective prevention and intervention strategies.

**Figure 1:**
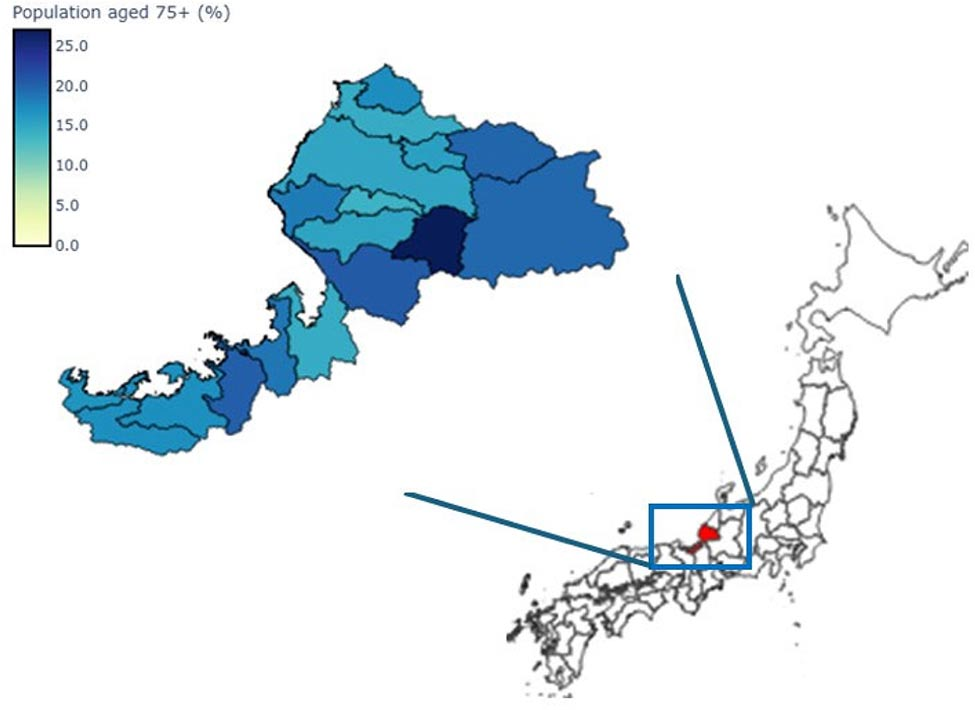
Proportion of the population aged 75 years and older in each municipality of Fukui prefecture

**Figure 2:**
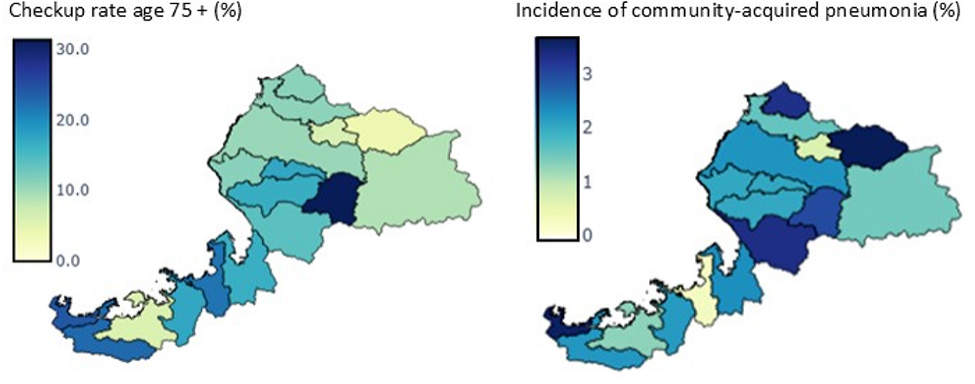
Health checkup participation rates and incidence rates of community-acquired pneumonia among adults aged 75 years and older in each municipality of Fukui prefecture

**Methods:**

This study is a retrospective cross-sectional study using the National Health Insurance and Later-Stage Elderly Healthcare System / Kokuho database (KDB), a nationwide repository encompassing Japanese-specific health checkup and health assessment data of older adults. We analyzed 22,753 participants aged 75 years and older who underwent health checkups from 2020 to 2021 in Fukui prefecture, Japan, to investigate the incidence of pulmonary infections within one year.

**Table 1:**
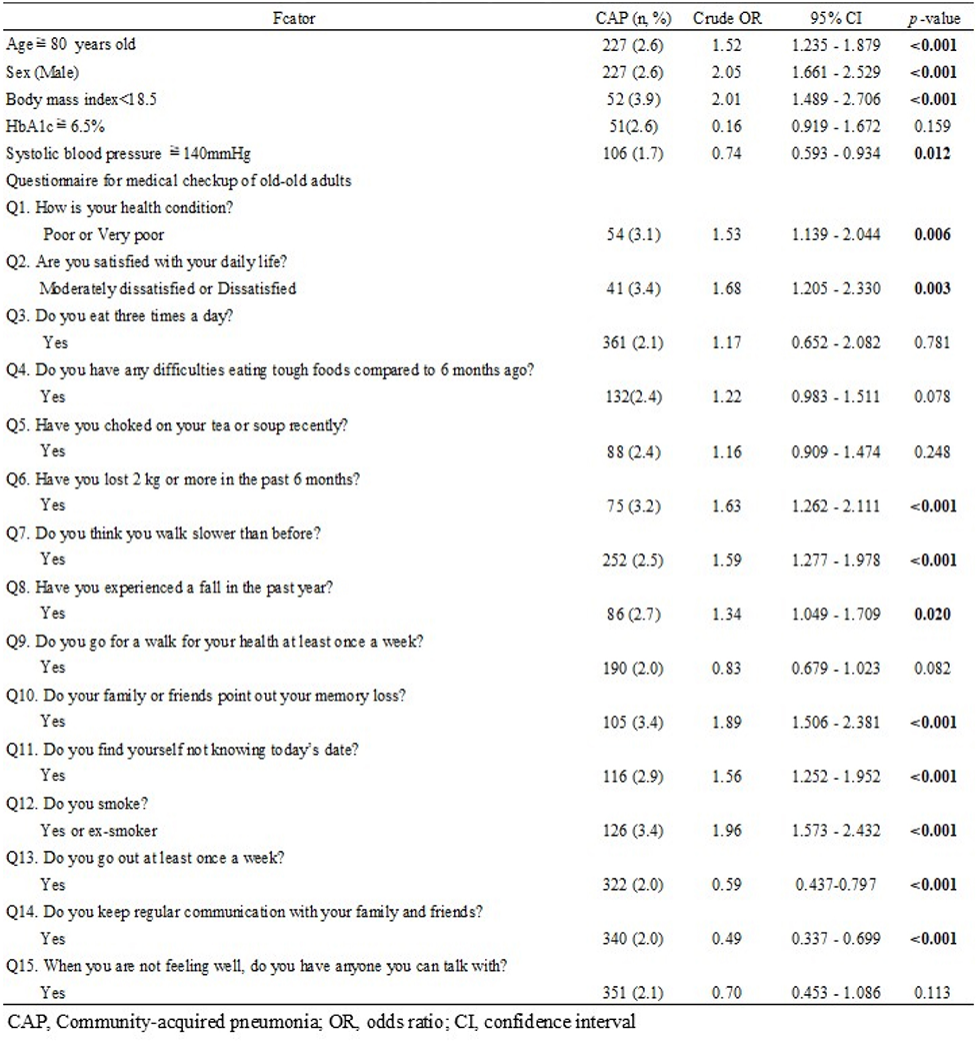
Univariate analysis of factors associated with community-acquired pneumonia among health checkup participants aged 75 years and older

**Table 2:**
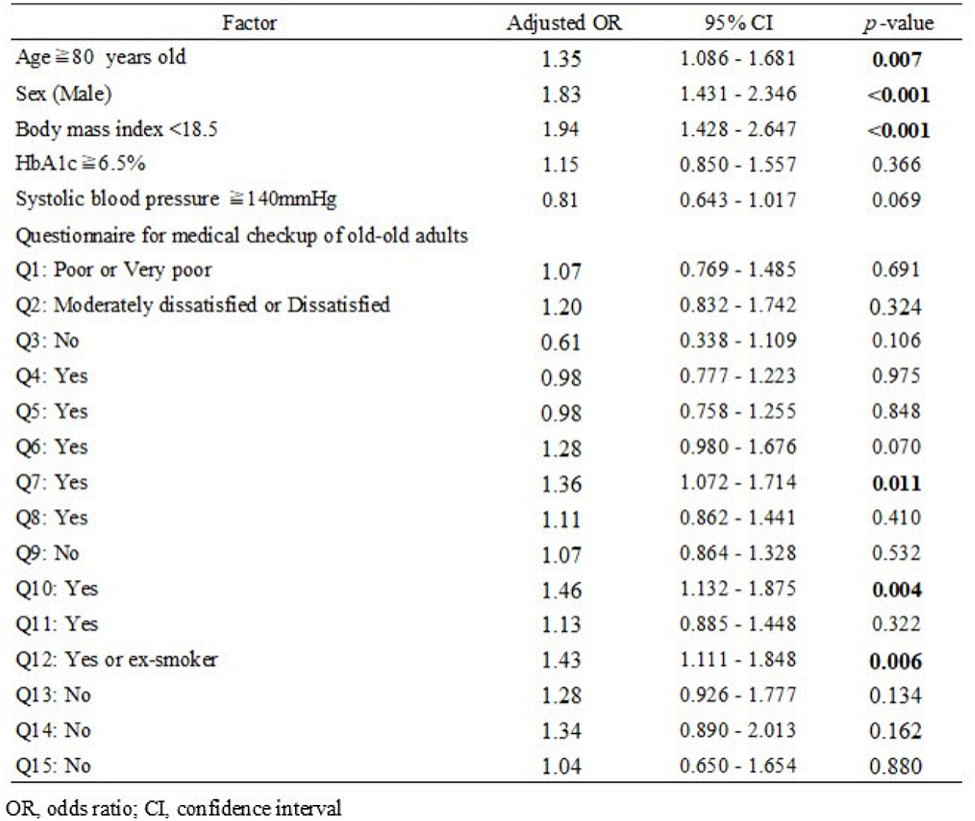
Multivariable analysis of factors associated with community-acquired pneumonia among health checkup participants aged 75 years and older

**Results:**

The incidence of pulmonary infections among health checkup participants was 2.2% (495/22753, including duplicate participants), and community-acquired pneumonia (CAP) accounted for 95.6% of these infections. While Fukui Prefecture has a relatively small population of 736,144 compared to other prefectures, a notable feature is the high proportion of adults aged 75 years and older across all municipalities (Figure 1). There were regional differences in the health checkup participation rate among those aged 75 and older, and municipalities with relatively lower checkup rates tended to show higher incidences of CAP (Figure 2). In the univariate analysis, significant associations with CAP were observed for 14 factors (Table 1). Multivariate logistic regression analysis revealed that the following factors were independently associated with an increased risk of CAP: age ≥80 years (OR: 1.35, p=0.007), male sex (OR: 1.83, p< 0.001), body mass index < 18.5 (OR: 1.94, p< 0.001), slower walking speed (Q7: OR: 1.36, 95%, p=0.011), memory loss reported by others (Q10: OR: 1.46, p=0.004), and smoking history (Q12: OR: 1.43, p=0.006) (Table 2).

**Conclusion:**

In late-stage older adults, CAP was shown to be influenced by various demographic, clinical, and functional factors. Identifying high-risk individuals through routine health checkups may contribute to early interventions and improved preventive strategies in the populations.

**Disclosures:**

All Authors: No reported disclosures

